# Enhanced ANGPTL2 expression in adipose tissues and its association with insulin resistance in obese women

**DOI:** 10.1038/s41598-018-32419-w

**Published:** 2018-09-18

**Authors:** Jimin Kim, Seul Ki Lee, Yeon Jin Jang, Hye Soon Park, Jong-Hyeok Kim, Joon Pio Hong, Yeon Ji Lee, Yoon-Suk Heo

**Affiliations:** 1 Department of Physiology, Cell Dysfunction Research Center, University of Ulsan College of Medicine, Seoul, Korea; 2 Department of Family Medicine, University of Ulsan College of Medicine, Seoul, Korea; 3 Department of Obstetrics and Gynecology, University of Ulsan College of Medicine, Seoul, Korea; 4 Department of Plastic Surgery, University of Ulsan College of Medicine, Seoul, Korea; 5 Department of Family Medicine, Inha University, College of Medicine, Incheon, Korea; 6 Department of General Surgery, Inha University, College of Medicine, Incheon, Korea

## Abstract

Angiopoietin-like protein 2 has been proposed to be a key mediator linking obesity and insulin resistance. However, no detailed study of ANGPTL2 expression in human adipose tissues has yet been reported. To investigate the pattern and regulation of ANGPTL2 expression in human adipose tissues in obesity and its related diseases, we recruited 32 non-diabetic and 13 type 2 diabetic obese women and 32 normal-weight women. ANGPTL2 mRNA was expressed at a similar level in visceral and subcutaneous adipose tissues. Adipose tissue ANGPTL2 mRNA was much higher in obese patients. Adipose tissue ANGPTL2 mRNA and serum ANGPTL2 levels showed strong associations with metabolic parameters associated with insulin resistance. In adipose tissue, ANGPTL2 mRNA was closely correlated with the expression of genes involved in inflammation and ER stress. ANGPTL2 mRNA was principally expressed in adipocytes, and its expression was markedly higher in the adipocyte but non-adipocyte fraction of obese adipose tissues. Culture of human adipocytes under conditions mimicking the microenvironment of obese adipose tissue (especially, increased ER stress) stimulated ANGPTL2 gene expression and secretion. In addition, co-culture of adipocytes and macrophages suggested that ANGPTL2 excessively produced by adipocytes, may contribute inflammation and remodeling in obese adipose tissues, thereby promoting insulin resistance.

## Introduction

Obesity is frequently associated with insulin resistance, a major pathogenic factor for type 2 diabetes. Conventionally, adipose tissue has been considered to be an energy storage organ. Recently, however, it has been recognized that adipose tissue is also a secretory organ releasing several biologically active molecules that exert autocrine, paracrine and endocrine effects^1^. In addition, chronic low-grade inflammation caused by dysfunction of adipose tissue plays a pivotal role in the development of insulin resistance in obesity^2,3^. Accordingly, the identification of the adipose tissue-derived factors linking adipose tissue inflammation and insulin resistance in obesity is of considerable interest.

Angiopoietin-like protein (ANGPTL) family is a recently identified group of proteins that are structurally similar to angiopoietin, which contain an N-terminal coiled-coil domain and a C-terminal fibrinogen-like domain^4–6^. ANGPTLs are also secreted but do not bind to the angiopoietin receptors Tie1 or Tie2^4,5,7^, suggesting that they act differently from angiopoietins. ANGPTL2 is a 57 kDa glycosylated protein member of this family^4^ and is mainly produced by adipose tissues^8^. ANGPTL2 expression in adipose tissues and its circulating level are both up-regulated in high fat diet-fed mice, and deletion of ANGPTL2 in these mice ameliorates adipose tissue inflammation as well as systemic insulin resistance^8^. Conversely, overexpression of ANGPTL2 in adipose tissue causes local inflammation and systemic insulin resistance in non-obese mice^8^. In db/db mice, adenovirus-mediated ANGPTL2 expression worsens insulin resistance and glucose intolerance, and promotes macrophage accumulation and proinflammatory M1 polarization in adipose tissues^9^. In human subjects who are overweight^10^, are obese^11^, or have type 2 diabetes^8^, serum ANGPTL2 concentration is also higher. In addition, circulating ANGPTL2 is closely associated with adiposity, systemic insulin resistance, and inflammation in healthy humans^8^. As a result, ANGPTL2 has been proposed as a key adipocyte-derived inflammatory mediator linking obesity to systemic insulin resistance^8^. However, studies in humans have mostly been conducted using only serum ANGPTL2 measurements. To our knowledge, no detailed study of ANGPTL2 expression in human adipose tissues has yet been reported.

The present study was undertaken to determine the pattern and regulation of ANGPTL2 expression in human adipose tissue in the context of obesity and associated diseases. We compared ANGPTL2 mRNA expression in the two separate fat depots, abdominal SAT and VAT, in obese women (with or without type 2 diabetes) and normal weight women. We investigated the associations of adipose tissue ANGPTL2 mRNA with metabolic parameters. We also measured serum ANGPTL2 level in these patients and explored the impact of weight loss on serum ANGPTL2 in obese patients who had undergone bariatric surgery. In addition, to understand better how ANGPTL2 expression is induced in obesity, we incubated differentiated human adipocytes in a series of culture environments mimicking obesity. Furthermore, we examined the role of ANGPTL2 in the crosstalk between adipocytes and macrophages using co-culture.

## Results

### Patient characteristics

The clinical characteristics of the non-diabetic obese, obese type 2 diabetic, and normal weight groups are shown in Supplementary Table 1. Their metabolic characteristics of the non-diabetic are summarized in Table 1. The non-diabetic obesity group was significantly younger than the other two groups. In comparison with the normal weight patients, obese patients displayed significantly higher BMI, blood pressure (BP), The homeostasis model assessment of insulin resistance index (HOMA-IR), and the circulating concentrations of insulin, triglyceride, high sensitivity C-reactive protein (hs-CRP) and leptin but significantly lower adiponectin. Abdominal computerized tomography (CT) also showed markedly higher total abdominal adipose tissue (TAT), VAT and SAT areas in obese patients than the control group. However, VAT area:SAT area ratio (VSR), an index of visceral obesity, was significantly higher only in diabetic obese patients.

**Table 1:** Metabolic parameters and abdominal fat distribution of the non-diabetic obese, obese type 2 diabetic, and normal weight control groups.

	Normal weight	Obesity	Obesity with diabetes
*n*	32	32	13
Age (yr)^a^	42.8 ± 6.2	32.8 ± 8.8^*^	45.3 ± 8.1^†^
BMI (kg/m^2^)^a^	22.5 ± 1.6	38.6 ± 5.2^*^	35.6 ± 6.5^*^
Systolic BP (mmHg)^a^	113 ± 15	137 ± 16^*^	135 ± 13^*^
Diastolic BP (mmHg)^b^	70 ± 2	83 ± 2^*^	84 ± 3^*^
Glucose (mM)^b^	5.6 ± 0.2	5.3 ± 0.1	11.9 ± 0.7^*,†^
Insulin (pM)^b^	26.4 ± 3.1	134.9 ± 12.2^*^	132.4 ± 25.9^*^
HOMA-IR^b^	1.1 ± 0.1	5.3 ± 0.5^*^	12.3 ± 2.7^*,†^
Total cholesterol (mg/dL)^a^	160 ± 36	182 ± 33	185 ± 52
HDL-cholesterol (mM)^a^	12.0 ± 2.6	12.3 ± 2.9	10.0 ± 2.2^*,†^
LDL-cholesterol (mM)^a^	2.6 ± 0.7	3.0 ± 0.8	2.9 ± 1.0
Triglyceride (mg/dL)^b^	73 ± 5	146 ± 21^*^	278 ± 69^*,†^
hs-CRP (mg/dL)^b^	0.08 ± 0.02	0.54 ± 0.09^*^	0.54 ± 0.12^*^
Leptin (ng/mL)^b^	5.7 ± 0.6	41.7 ± 3.0^*^	22.2 ± 3.0^*,†^
Adiponectin (μg/mL)^b^	6.7 ± 0.6	2.4 ± 0.3^*^	3.4 ± 1.4^*^
HbA1c (%)^b^	N/A	5.8 ± 0.1	10.0 ± 0.6^†^
*Abdominal CT*			
TAT area (cm^2^)^a^	227 ± 73	654 ± 154^*^	587 ± 182^*^
VAT area (cm^2^)^a^	67 ± 30	141 ± 60^*^	223 ± 50^*,†^
SAT area (cm^2^)^a^	159 ± 60	513 ± 151^*^	363 ± 155^*,†^
VSR^a^	0.48 ± 0.25	0.31 ± 0.22^*^	0.67 ± 0.20^*,†^

^a^Data are shown as the mean ± SD. ^b^The data were log-transformed for statistical analysis. Data are shown as the mean ± SE on the original (back-transformed) scale. *p < 0.05 *vs*. control, ^†^*p* < 0.05 *vs*. obesity group analyzed using one-way ANOVA followed by Tukey’s *post hoc* test. N/A, not available.

### Higher expression of ANGPTL2 mRNA in adipose tissues of obese and diabetic patients

**Figure 1:**
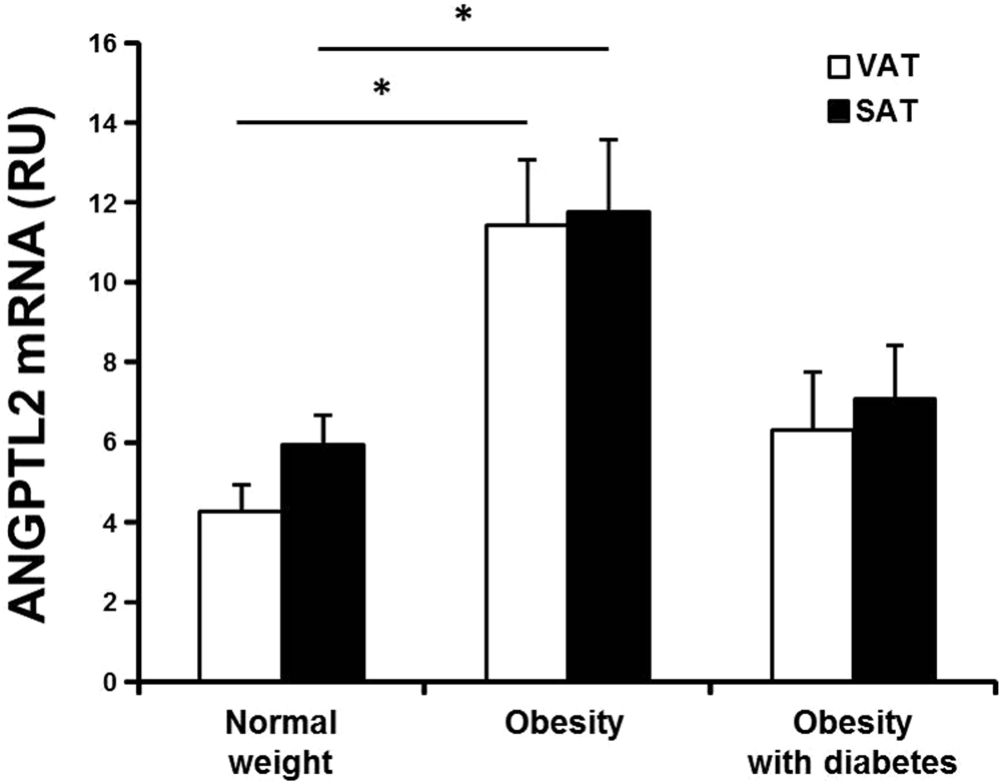
ANGPTL2 mRNA expression in human adipose tissues. ANGPTL2 mRNA in the VAT and SAT of the normal-weight control (n = 32), non-diabetic obesity (n = 31), and obesity with type 2 diabetes (n = 14) groups. β-actin was used as the reference gene; ANGPTL2 mRNA levels are presented in relative units (RU). Data are log-transformed for statistical analysis and shown as the mean ± SE on the original (back-transformed) scale. *p < 0.05 *vs*. the corresponding fat depot in the control group by ANOVA with Tukey’s test.

For all three groups, VAT and SAT were not different in terms of mean ANGPTL2 mRNA (Fig. 1), and there was a strong correlation between ANGPTL2 mRNA in VAT and SAT (*r* = 0.499; *p* < 0.001; *n* = 77), indicating that ANGPTL2 mRNA is expressed at a similar level in the two fat depots. However, we found that ANGPTL2 mRNA in both fat depots was significantly higher in non-diabetic obese patients than normal weight patients. In diabetic obese patients, adipose tissue ANGPTL2 mRNA also showed a trend of increment, but without statistical significance.

**Figure 2:**
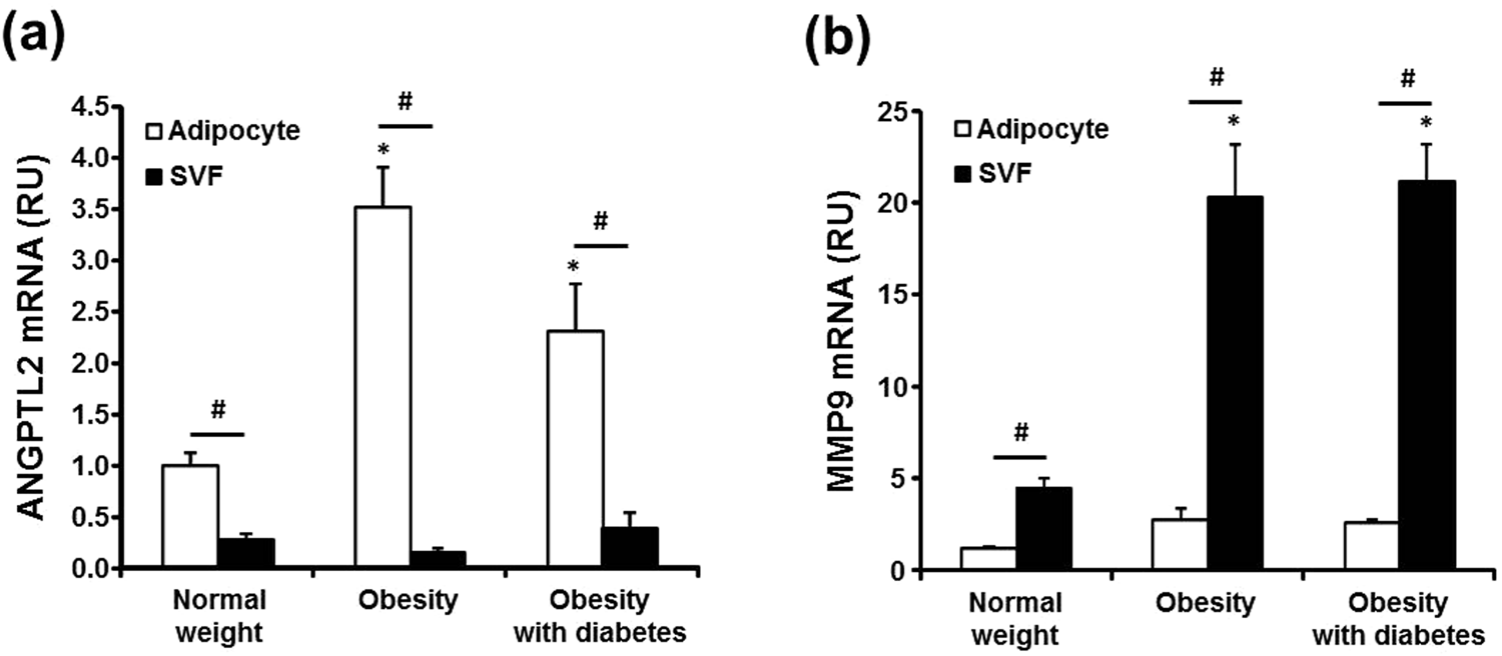
ANGPTL2 and MMP9 mRNA levels in adipocyte and the SVF obtained from adipose tissues. (**a**,**b**) VAT of normal-weight women (*n* = 8) who received benign gynecological surgery and non-diabetic obese women (*n* = 8) and obese women with type 2 diabetes (*n* = 4) who received RYGB surgery, were removed during surgery and digested with collagenase to separate floating adipocytes and non-floating SVF. β-actin was used as the reference gene. *p < 0.05 *vs*. control group by ANOVA with Tukey’s test, ^#^*p* < 0.01 *vs*. adipocyte by paired *t*-test.

Next, we determined the mRNA expression of ANGPTL2 (Fig. 2a) and MMP9 (Fig. 2b), a potential target gene of ANGPTL2^12^, in adipocytes and stromal/vascular cell fraction (SVF) from adipose tissue. ANGPTL2 mRNA expression was much higher in adipocytes than SVF in all three groups (Fig. 2a). Moreover, adipocyte ANGPTL2 mRNA was noticeably higher in obese patients with or without diabetes than in normal weight subjects. Conversely, ANGPTL2 mRNA expression in SVF was not different among the groups. In contrast to ANGPTL2, MMP9 mRNA expression was predominantly in SVF (Fig. 2b). SVF MMP9 mRNA was much higher in obese patients than normal weight patients, whereas there was no significant difference in adipocyte MMP9 mRNA amongst the groups.

### Correlations between adipose tissue ANGPTL2 mRNA and metabolic parameters

In the entire study cohort (*n* = 77), we assessed the correlation between adipose tissue ANGPTL2 mRNA and metabolic parameters (Table 2). VAT ANGPTL2 mRNA correlated significantly and positively with BMI, BP, HOMA-IR, insulin, LDL-cholesterol, triglyceride, hs-CRP, and leptin, and negatively with serum adiponectin; SAT ANGPTL2 mRNA was significantly positively correlated with HOMA-IR, insulin, triglyceride, and leptin. However, all the significant correlations between adipose tissue ANGPTL2 mRNA and metabolic parameters disappeared after adjustment for BMI. ANGPTL2 mRNA was not correlated with adipocyte size in VAT or SAT (Supplementary Fig. 1).

**Table 2:** Correlations between metabolic parameters and adipose tissue ANGPTL2 mRNA or serum ANGPTL2 in study participants.

	ANGPTL2 mRNA		Serum ANGPTL2	
	VAT	SAT		
	*r*	*r*	*r*	*r* ^*a*^
BMI	**0**.354^*^	0.138	**0**.434^*^	
Age	−0.219	−0.189	−0.173	0.096
Systolic BP	**0**.269^*^	0.147	**0**.359^*^	0.205
Diastolic BP	**0**.297^*^	0.153	**0**.384^*^	0.210
Glucose	−0.036	0.002	**0**.350^*^	**0**.430^*^
Insulin	**0**.344^*^	**0**.261^*^	**0**.433^*^	0.178
HOMA-IR	**0**.300^*^	**0**.235^*^	**0**.486^*^	**0**.319^*^
Total cholesterol	0.133	0.086	**0**.302^*^	0.016
HDL-cholesterol	0.007	−0.080	0.016	−0.242
LDL-cholesterol	**0**.251^*^	0.190	**0**.242^*^	−0.111
Triglyceride	**0**.268^*^	**0**.259^*^	**0**.471^*^	**0**.325^*^
hs-CRP	**0**.313^*^	0.202	**0**.477^*^	0.130
Leptin	**0**.351^*^	**0**.272^*^	**0**.361^*^	0.128
Adiponectin	−**0**.305^*^	−0.147	0.178	−0.138
VSR	−0.245	−0.183	0.189	**0**.271^*^

*n* = 77, *r* = Pearson correlation coefficient; *r*^a^ = Correlation coefficient after adjustment for BMI; *P < 0.05.

### Association between ANGPTL2 mRNA expression and the expression of other gene transcripts in human adipose tissue

We also examined the correlation between ANGPTL2 mRNA expression and that of genes encoding inflammatory cytokines (TNF-α and IL-1β), a macrophage marker (CD68), endoplasmic reticulum (ER) stress markers (TRIB3 and CHOP), adipokines affecting insulin sensitivity (leptin and adiponectin), and MMP9 in each fat depot (Table 3). In both VAT and SAT, ANGPTL2 mRNA did not correlate with adiponectin mRNA; however, ANGPTL2 mRNA significantly positively correlated with most of the gene transcripts we assayed.

**Table 3:** Correlations between the mRNA expression of ANGPTL2 and that of other genes in the adipose tissue depots of study participants.

	VAT		SAT	
	*r*	*p*	*r*	*p*
Adiponectin	−0.116	0.317	0.070	0.544
Leptin	**0**.**307**	**0**.**007**	**0**.**315**	**0**.**005**
TNF-α	**0**.**555**	**<0**.**001**	**0**.**271**	**0**.**017**
IL-1β	0.132	0.251	**0**.**255**	**0**.**025**
CD68	**0**.**318**	**0**.**005**	**0**.**322**	**0**.**004**
TRIB3	**0**.**317**	**0**.**005**	**0**.**253**	**0**.**026**
CHOP	**0**.**404**	**<0**.**001**	**0**.**306**	**0**.**007**
MMP9	**0**.**255**	**0**.**025**	**0**.**259**	**0**.**023**

*n* = 77, *r* = Pearson correlation coefficient.

### High serum ANGPTL2 in obese patients and its reduction by Roux-en-Y gastric bypass (RYGB)-induced weight loss

**Figure 3:**
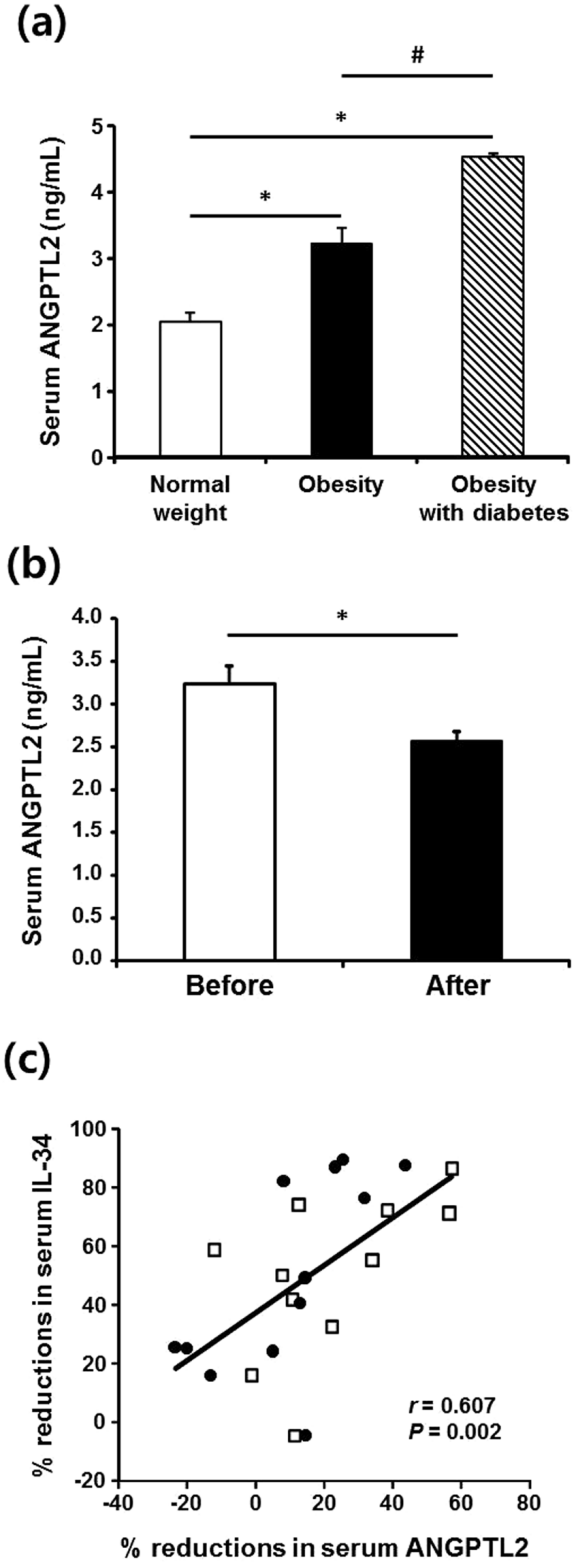
Serum ANGPTL2 concentrations and ANGPTL2 mRNA expression in human. (**a**) Serum ANGPTL2 concentrations in patients with obesity (n = 31), obesity and type 2 diabetes (n = 14), and normal-weight control patients (n = 32). Data are log-transformed for statistical analysis and shown as the mean ± SE on the original (back-transformed) scale. *p < 0.01 vs. control, ^#^*p* < 0.01 vs. obesity group according to ANOVA and the Tukey’s test. (**b**) Significant reduction in serum ANGPTL2 concentration following RYGB surgery in obese patients. Serum ANGPTL2 concentration and metabolic parameters were measured before and 5–9 months (average 7.3 months) after surgery in a subgroup of obese patients (*n* = 23). *p < 0.01 vs. before surgery according to paired *t*-test. (**c**) Close association between percentage reduction in serum ANGPTL2 concentrations and percentage reduction in serum IL-34 following the surgery. Percent reduction in serum ANGPTL2 was calculated as [(serum ANGPTL2 before surgery – serum ANGPTL2 after surgery) ÷ serum ANGPTL2 before surgery] × 100. The correlation coefficient (*r*) between changes in measurements was calculated using Spearman’s correlation.

Serum ANGPTL2 was significantly higher in non-diabetic obese patients than normal weight patients; in obese patients, it was significantly higher when type 2 diabetes was also present (Fig. 3a). Next, we examined the correlation between serum ANGPTL2 and metabolic parameters in the entire study cohort (Table 2). Serum ANGPTL2 was significantly positively correlated with BMI, BP, HOMA-IR, glucose, insulin, total cholesterol, LDL-cholesterol, triglyceride, hs-CRP, and leptin. When adjusted for BMI, serum ANGPTL2 showed significant positive correlations with glucose, triglyceride, HOMA-IR, and VSR. In addition, RYGB significantly reduced serum ANGPTL2 (Fig. 3b), alongside marked weight loss and improvement in metabolic parameters (Supplementary Table 2). In these patients, the decrease in serum ANGPTL2 was strongly positively correlated with the decrease in serum interleukin (IL)-34 (Fig. 3c), a marker of chronic inflammation^13^; it also showed a positive association with the decrease in hs-CRP (*r* = 0.400; *P* = 0.065; *n* = 23; data not shown).

### Regulation of ANGPTL2 expression and secretion in human adipocytes

**Figure 4:**
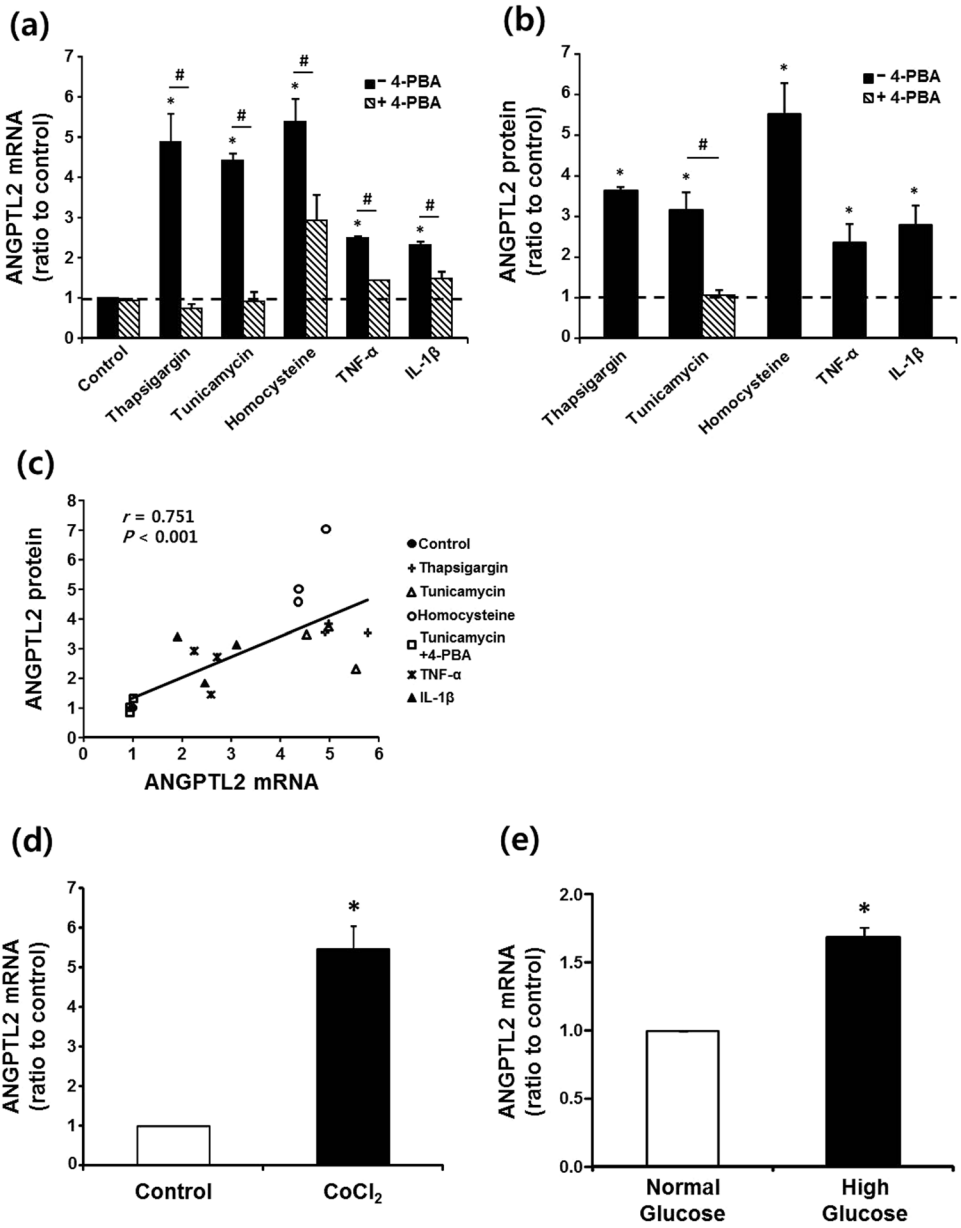
Induction of ANGPTL2 in human adipocytes. (**a**,**b**) Up-regulation of ANGPTL2 by ER stressors and proinflammatory cytokines. Fully differentiated human adipocytes (*n* = 3) were incubated in serum-free media for 24 hours with or without (control) various stressors such as thapsigargin (500 nM), tunicamycin (2 μg/mL), homocysteine (4 mM), TNF-α (10 ng/mL), and IL-1β (10 ng/mL). Effect of chemical chaperon was examined by adding 4-PBA (500 μM) to the culture media 2 hours prior to the treatment with stressors. ANGPTL2 mRNA level was measured by qPCR using 36B4 as the reference gene (**a**) and secreted ANGPTL2 protein was assessed by measuring its concentration in the culture media by ELISA (**b**). *p < 0.05 *vs*. control by ANOVA with Tukey test, ^#^*p* < 0.05 *vs*. without 4-PBA by paired *t*-test. Dotted line represents ANGPTL2 mRNA or protein level at control state without stimulation. (**c**) Correlation between ANGPTL2 mRNA expression and ANGPTL2 protein secretion in differentiated human adipocytes. The correlation coefficient (*r*) between the measurements in experiments described above in A & B was calculated using Spearman’s correlation. (**d**,**e**) Effects of hypoxia or glucose on ANGPTL2 mRNA expression. (**d**) Differentiated human adipocytes were incubated in serum-free media for 24 hours with or without (control) a chemical hypoxic inducer CoCl_2_ (100 μM). *p < 0.05 *vs*. control by paired *t*-test. (**e**) Cells were incubated in the media containing either 5.5 mM (normal glucose) or 25 mM glucose (high glucose). *p < 0.05 *vs*. the normal glucose condition by paired *t*-test.

To understand the mechanisms underpinning the higher ANGPTL2 expression in adipocytes from obese adipose tissue, we incubated differentiated human adipocytes in culture conditions mimicking the local microenvironment of obese adipose tissue (Fig. 4). Consistent with the findings in 3T3-L1 adipocytes^8^, the expression of ANGPTL2 mRNA was markedly enhanced by ER stress inducers (thapsigargin, tunicamycin, and homocysteine) and proinflammatory cytokines (TNF-α and IL-1β) in human adipocytes (Fig. 4a). Concomitant addition of 4-phenylbutyrate (4-PBA) significantly reduced or completely prevented the increases in ANGPTL2 mRNA. These stressors also increased ANGPTL2 protein secretion. In addition, and as expected, 4-PBA completely blocked tunicamycin-induced ANGPTL2 protein secretion (Fig. 4b). In these experiments, there was a strong correlation between ANGPTL2 mRNA expression and secreted ANGPTL2 protein (Fig. 4c), indicating that differences in ANGPTL2 mRNA in human adipocytes accurately reflect differences in secreted ANGPTL2 protein. Additionally, ANGPTL2 mRNA was markedly increased by CoCl_2_, a mimic of hypoxia (Fig. 4d). Likewise, incubation of the cells in high glucose medium significantly further increased ANGPTL2 mRNA over that in normal glucose medium (Fig. 4e). Thus, our data indicate that altered local environment, such as increased ER stress might be an important cause of the higher ANGPTL2 production by adipocytes in obese adipose tissue.

### Involvement of ANGPTL2 in the crosstalk between adipocytes and macrophages in adipose tissue

**Figure 5:**
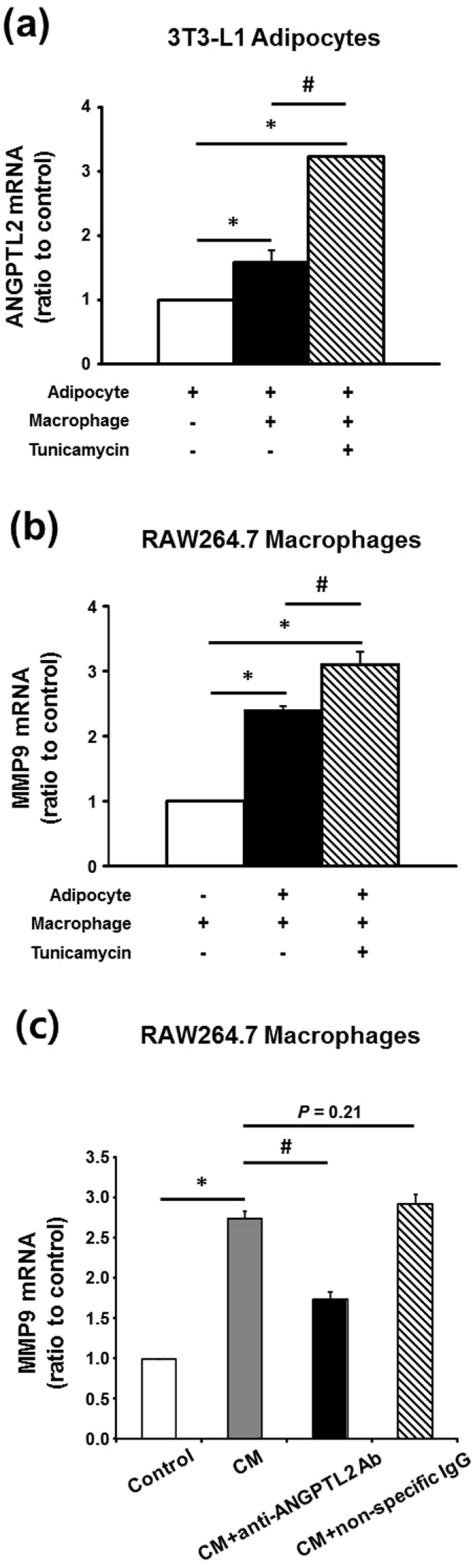
Induction of ANGPTL2 and MMP9 mRNA expression by co-culture of adipocytes and macrophages and reduction of MMP9 mRNA expression by ANGPTL2 blockade. (**a**,**b**) Differentiated 3T3-L1 adipocytes were co-cultured for 24 hours with RAW264.7 macrophages using transwell system. Measurement of ANGPTL2 mRNA expression in differentiated adipocytes (**a**) and MMP9 mRNA expression in macrophages (**b**) was performed by qPCR (n = 3). Incubation with tunicamycin (2 μg/mL) was conserved in both of cells for 16 hours. *p < 0.05 *vs*. each of cells alone, ^#^*p* < 0.05 *vs*. co-cultured cells. The data were analyzed by ANOVA with Tukey test. (**c**) Induction of MMP9 mRNA by adipocyte-conditioned media (CM) and partially reduction of MMP9 mRNA expression by anti-ANGPTL2 antibody (4 μg/mL). RAW264.7 macrophages were incubated for 16 hours in the presence of CM, CM+ anti-ANGPTL2 Ab, or CD+ non-specific IgG (n = 3). *p < 0.05 *vs*. control, ^#^*p* < 0.05 *vs*. CM by ANOVA with Tukey test.

ANGPTL2 mRNA was highly expressed in adipocytes (Fig. 2a), whereas MMP9 mRNA was enriched in the SVF (Fig. 2a). Consistent with this, MMP9 is reported to be prominently expressed by macrophages in human adipose tissue^14^. Therefore, we tested the hypothesis that in obese adipose tissues, excessive ANGPTL2 production by adipocytes up-regulates MMP9 in macrophages by co-culturing 3T3-L1 adipocytes and RAW264.7 macrophages (Fig. 5). In 3T3-L1 cells, ANGPTL2 mRNA was induced during adipogenesis; however, when adipocytes were cultured for a prolonged period to induce hypertrophy, ANGPTL2 mRNA was not increased any further, despite additional lipid accumulation (Supplementary Fig. 2). Therefore, for co-culture experiments, we employed fully differentiated 3T3-L1 adipocytes with tunicamycin to mimic the microenvironment of obese adipose tissue with ER stress. Tunicamycin markedly increased ANGPTL2 mRNA in 3T3-L1 adipocytes cultured alone, whereas tunicamycin alone did not affect MMP9 mRNA expression in RAW264.7 macrophages (Supplementary Fig. 3). When the two cell types were co-cultured, ANGPTL2 mRNA expression in adipocytes was significantly higher, and was higher still in the presence of tunicamycin (Fig. 5a). In macrophages, MMP9 mRNA was significantly up-regulated when the cells were co-cultured with adipocytes, and it was also further enhanced when tunicamycin was added (Fig. 5b). As expected, tumor necrosis factor (TNF)-α and IL-1β mRNA showed similar expression patterns in these co-cultured macrophages (Supplementary Fig. 4). These findings suggest that the increase in MMP9 mRNA expression as well as TNF-α and IL-1β mRNA expression in macrophages generated by co-culturing with adipocytes, especially in the presence of an inducer of ER stress, might be caused by ANGPTL2 secreted by adipocytes. To test this possibility, we utilized adipocyte-conditioned media (CM) and an anti-ANGPTL2 antibody. Adipocyte-CM was prepared by collecting the supernatant from the 3T3-L1 adipocytes cultured in the presence of tunicamycin. In RAW264.7 macrophages, adipocyte-CM significantly increased MMP9 mRNA by 2.7-fold whereas adipocyte-media (made in the same way as adipocyte-CM but with no tunicamycin) did not (Supplementary Fig. 5). As shown in Fig. 5c, concomitant treatment with anti-ANGPTL2 antibody prevented about 60% of MMP9 mRNA expression induced by adipocyte-CM, but the addition of non-specific IgG did not show any effect.

## Discussion

ANGPTL2 participates in adaptive inflammation and tissue reconstruction, maintaining tissue homeostasis; however, an excessive ANGPTL2 signal, resulting from prolonged stress, can promote chronic inflammation and irreversible tissue remodeling^12^. Accordingly, ANGPTL2 is implicated in many chronic diseases, including atherosclerosis^8^, diabetes^8,15^, cancer^16^, and other metabolic disorders^17^. Intriguingly, ANGPTL2 is abundantly expressed in adipose tissue^8^. Through studies employing obese or diabetic animals^8,9^, ANGPTL2 has been shown to play an important role in adipose tissue inflammation and insulin resistance. However, data regarding ANGPTL2 expression in human adipose tissue are extremely limited, and to our knowledge, studies in humans have mostly measured serum ANGPTL2.

In all three groups of our patients, ANGPTL2 mRNA was abundantly expressed in VAT and SAT at a similar level, whereas it is more highly expressed in epididymal adipose tissue than SAT in mice^8^. VAT has previously been proposed to be a major source of circulating ANGPTL2 in obese subjects, based on a positive association between circulating ANGPTL2 and VAT accumulation^8^; however, our direct measurements of adipose tissue ANGPTL2 mRNA suggest equal contributions of the two fat depots to serum ANGPTL2 instead. As expected, adipose tissue ANGPTL2 mRNA expression was much higher in obese patients. ANGPTL2 mRNA in adipose tissues, especially in VAT, showed significant associations with metabolic parameters influenced by inflammation, dyslipidemia, and insulin resistance; however, the significant correlations were abolished after adjustment for BMI. It thus appears that ANGPTL2 mRNA up-regulation in human adipose tissue is closely related to the metabolic derangements associated with greater adiposity. Moreover, in each fat depot, ANGPTL2 mRNA strongly and positively correlated with the expression of genes involved in inflammation and ER stress. These results support the notion that an altered local microenvironment increases ANGPTL2 expression in obese adipose tissue^8^.

Adipocytes have been thought to be the major source of ANGPTL2 in obese adipose tissue. However, this required clarification, because not only adipocytes, but also other cell types, including macrophages and endothelial cells, produce ANGPTL2^8,18–20^. To this end, we fractionated adipose tissue and found that ANGPTL2 mRNA was enriched in the adipocyte fraction rather than the SVF, agreeing with the previous immunohistochemical analysis of human adipose tissue that detected ANGPTL2 in adipocytes^8^. ANGPTL2 mRNA in the adipocyte fraction was noticeably higher in obese patients, whereas that in the SVF (composed of various cell types, including macrophages, endothelial cells, and preadipocytes) was not different from that in normal weight patients. Thus, our data confirm that adipocytes are the main cells responsible for the over-production of ANGPTL2 in human adipose tissue during obesity.

We next asked what stimulates ANGPTL2 production by adipocytes during obesity. ANGPTL2 secretion and mRNA expression by differentiated human adipocytes was markedly enhanced by incubating the cells under conditions mimicking the microenvironment of obese adipose tissue. Conversely, adipocyte hypertrophy *per se*, resulting from over-accumulation of lipids, might also increase ANGPTL2 expression in obesity, because ANGPTL2 expression is induced during adipogenesis^8^. However, our observations do not support this possibility, because no correlation was observed between adipocyte size and ANGPTL2 mRNA expression in VAT or SAT, and the over-accumulation of lipids in 3T3-L1 adipocytes did not increase ANGPTL2 mRNA. Hence, in obese adipose tissue, the increase in ANGPTL2 production by adipocytes appears to be mainly due to the change in the microenvironment associated with adipocyte hypertrophy. Various changes in this microenvironment can promote ER stress, including inflammation and hypoxia^21^. Indeed, treatment of human adipocytes with a chemical chaperone at least partially inhibited ER stressor- and inflammatory cytokine-induced ANGPTL2 mRNA expression. Thus, our data strongly support the contention that ER stress is an important cause of the higher ANGPTL2 expression by adipocytes in obese adipose tissue^8^. We are now performing further studies to identify the specific ER stress signaling pathways involved in ANGPTL2 transcription in adipocytes.

Intercellular communication between adipocytes and macrophages *via* cell-cell contact or humoral factors is important in the adipose tissue dysfunction associated with obesity. In adipose tissue, ANGPTL2 is mainly produced by adipocytes, while proinflammatory cytokines and MMP9, putative targets of ANGPTL2^12^, are abundantly expressed in macrophages. We thus hypothesized that, in obese adipose tissue, the excessive ANGPTL2 production by adipocytes up-regulates the expression of these target molecules in local macrophages. To test this hypothesis, we replicated the conditions in obese adipose tissue by co-culturing 3T3-L1 adipocytes and RAW264.7 macrophages in the presence of the ER stressor tunicamycin. As a result, alongside the marked increase in ANGPTL2 mRNA expression in adipocytes, the mRNA expression of TNF*-α*, IL-1β, and MMP9 was higher in the macrophages. In addition to our data, there is already substantial evidence supporting a stimulatory effect of ANGPTL2 on the expression of inflammation-related genes in macrophages. Overexpression of ANGPTL2 in non-obese mice and db/db mice increases mRNA expression of proinflammatory cytokines in adipose tissue^8,9^. As more direct evidence, treatment of the human macrophage-like cell line THP-1 and murine peritoneal macrophages with recombinant ANGPTL2 increases the transcription and secretion of chemoattractants and proinflammatory cytokines, such as monocyte chemotactic protein-1, TNF-α, and IL-1β. In the current study, we thus focused on the effect of adipocyte-derived ANGPTL2 on MMP9 mRNA expression in macrophages^9,22^. MMP9 directly degrades extracellular matrix (ECM) proteins and activates cytokines and chemokines to regulate tissue remodeling^23^. In addition, importantly, MMP9 mRNA expression is higher in the adipose tissue of obese patients, and is correlated with insulin resistance^24,25^. Conditioned medium from ANGPTL2-oversecreting cells increased MMP9 mRNA expression in macrophages, and simultaneous addition of anti-ANGPTL2 antibody to the medium significantly inhibited this effect. Furthermore, ANGPTL2 mRNA significantly correlated with TNF-α, IL-1β, and MMP9 mRNA in VAT and SAT, further supporting our hypothesis. Taken altogether, it is suggested that ANGPTL2 mediates crosstalk between adipocytes and macrophages, thereby promoting inflammation and ECM remodeling in obese adipose tissue.

As expected, serum ANGPTL2 was high in our obese patients. However, unlike adipose tissue ANGPTL2 mRNA expression, serum ANGPTL2 was higher when type 2 diabetes was present in addition to obesity. Because the changes in ANGPTL2 mRNA expression appeared accurately to reflect alterations in ANGPTL2 secretion, the discrepancy between the two measurements in obese patients associated with type 2 diabetes occurred to a surprisingly great extent. However, our observation is consistent with a previous report of higher serum ANGPTL2 in obese subjects with insulin resistance than in metabolically healthy obese subjects^11^. It was recently shown that, in severely obese patients, serum ANGPTL2 is higher when the patients also have type 2 diabetes, coronary artery disease, and dyslipidemia^26^. While associations of adipose tissue ANGPTL2 mRNA with metabolic parameters disappeared after adjusting for BMI, serum ANGPTL2 strongly correlated with the degree of hyperglycemia, hypertriglyceridemia, insulin resistance, and visceral obesity after adjustment for BMI. These data thus suggest that, in addition to expanded adipose tissue, other tissues contribute to elevated circulating ANGPTL2 in obese patients, especially when complications including in type 2 diabetes were present. Further studies are required to clarify this issue.

After RYGB, serum ANGPTL2 was significantly lower, and there were improvements in BMI and metabolic parameters. In these patients, the reduction in ANGPTL2 was positively correlated with reductions in indices of chronic inflammation, indicating an intimate relationship between serum ANGPTL2 and inflammation. While we were preparing this manuscript, a decrease in circulating ANGPTL2 was reported after weight loss induced by another type of bariatric surgery, biliopancreatic diversion with duodenal switch, in severely obese patients^26^. In this study, the reduction in serum ANGPTL2 was associated with an improved inflammatory and cardiometabolic profile, rather than changes in body weight and VAT accumulation. Reductions in circulating ANGPTL2 have also been reported following PPARγ agonist treatment of obese diabetic men^8^, as well as after lifestyle intervention-induced weight loss in overweight individuals^10^.

There are notable limitations to the present study. First, it is difficult to determine the causality of the observed relationships, due to the cross-sectional study design. Second, the number of subjects in the study groups was small, and consequently the study was not powerful enough to discern the confounding factors in our analysis. Third, our subject groups were not age-matched: the non-diabetic obesity group was significantly younger than the other two groups. Although the relationship between ANGPTL2 expression and age is largely unknown, it is possible that like most factors associated with chronic diseases, ANGPTL2 expression can increase with aging. If so, younger age in nondiabetic obese group can cause to underestimate the influence of obesity on the adipose tissue ANGPTL2 mRNA or serum ANGPTL2 levels. Finally, we used HOMA-IR instead of glucose clamp, the gold standard for measuring whole-body insulin sensitivity. However, HOMA-IR measurements correlate well with results obtained using the glucose clamp method^27^.

In conclusion, the present study shows, for the first time to our knowledge, the pattern and regulation of ANGPTL2 expression in human adipose tissue in the context of obesity and type 2 diabetes. Parallel measurement of ANGPTL2 mRNA in VAT and SAT and serum ANGPTL2 revealed not only their inter-relationships, but also their close associations with adiposity, inflammation, and insulin resistance. Our data from a combination of *in vivo* and *in vitro* cell culture experiments support the contention that ANGPTL2 production by adipocytes is increased by alterations in the microenvironment, especially ER stress, which are caused by obesity. In addition, it suggests that ANGPTL2 produced by adipocytes up-regulates MMP9 and proinflammatory cytokine production in macrophages, thereby promoting adipose tissue inflammation, remodeling, and systemic insulin resistance.

## Methods

### Study subjects and adipose tissue sampling

The study cohort consisted of 32 obese women (BMI ≥ 30 kg/m^2^) without diabetes, 13 obese women with type 2 diabetes, and 32 normal weight women (18.5 kg/m^2^ < BMI ≤ 25 kg/m^2^) (total 77 women). All obese patients underwent laparoscopic RYGB at the Obesity Center of the Inha University Hospital (Incheon, Korea). Four days before surgery, patients were admitted and underwent routine physical examinations, biochemical analyses after fasting, and abdominal CT. A subgroup of obese patients (*n* = 23: 12 non-diabetic and 11 diabetic obese patients) was followed up 5–9 months after RYGB, when metabolic parameters and serum ANGPTL2 concentration were assessed. Additionally, normal weight women who were undergoing elective abdominal surgery for benign conditions in the Gynecology Unit of the Asan Medical Center (Seoul, Korea) were recruited as controls. Women with evidence of malignancy or severe hepatic or renal disease, and those who were pregnant or lactating, were excluded. All subjects provided written informed consent at enrollment. The study protocol was approved by the Institutional Review Boards of the Asan Medical Center and the Inha University Hospital. All applicable institutional regulations regarding the ethical use of human volunteers were followed. Some of the samples obtained from these patients were analyzed in previous reported studies^13,28^.

BP, anthropometric measurements were performed as previously described^28^. During surgery, 2–5 g samples of VAT and SAT were removed as previously described^13,28^.

### Measurements of metabolic variables and serum ANGPTL2 concentration

Blood samples were obtained 3 days after the discontinuation of medication and following a 12 hour fast, and plasma and serum were immediately separated by centrifugation. Circulating concentrations of glucose, insulin, cholesterol, triglyceride, hs-CRP, IL-34, leptin, and adiponectin were measured, as previously described^13^. HOMA-IR was calculated as previously described^27^. Serum concentrations of adiponectin (AdipoGen, Incheon, Korea), leptin (R&D Systems, Abingdon, Oxfordshire, UK), IL-34 (Wuhan EIAab Science Co., Ltd, Wuhan, China), and ANGPTL2 (IBL, Gunma, Japan) were measured using commercial enzyme-linked immunosorbent assay (ELISA) kits. The assay sensitivity for ANGPTL2 was 0.01 ng/mL, and the intra- and interassay coefficients of variation were 3.9–5.9% and 6.3–10.5%, respectively.

### Estimation of abdominal fat distribution

Abdominal fat distribution was assessed using CT, as previously described^28^. The areas of TAT, VAT, and SAT were assessed using cross-sectional scans that were centered on the L4–L5 vertebral disc space. In addition, VSR, an index of visceral obesity^29,30^, was calculated.

### Fractionation of adipocytes and stromal/vascular cells

A subgroup of study subjects participated in this study: 8 normal-weight women, 8 obese women without diabetes and 4 obese women with type 2 diabetes. During surgery, 10–15 g of VAT was removed and the adipocyte and SVF was separated, as previously described^28^.

### Real-time quantitative RT-PCR

For the measurement of mRNA expression, RNA isolation and real-time quantitative RT-PCR (qPCR) were performed, as described previously^28^; the primer sequences are shown in Supplementary Table 3.

### Adipocyte size measurement

Adipocyte size was estimated using the mean cross-sectional area of adipocytes in hematoxylin and eosin (H&E)-stained sections of adipose tissue, as previously described^31^. Paired samples of VAT and SAT were obtained from a subgroup of subjects. On each H&E slide, digital photomicrographs (x100) were acquired in two separate areas (containing 500–1,000 adipocytes). The cross-sectional areas of adipocytes were estimated using Image Pro plus software (Media Cybernetics, version 4.5.0.29, Rockville, MD, USA) in an average of two separate areas from each slide.

### Isolation and differentiation of human preadipocytes and treatment of differentiated adipocytes

We separately recruited nine women (BMI = 23.5 ± 2.2 kg/m^2^; age = 46.8 ± 7.4 years) undergoing breast reconstruction surgery. Adipose tissues were harvested from discarded subcutaneous tissues that were obtained from the transverse rectus abdominis musculocutaneous flap during breast reconstruction. All protocols were approved by the ethics committee of Asan Institute for Life Sciences (Seoul, Korea), and all patients provided written informed consent. The isolation of human preadipocytes and differentiation to adipocytes were performed as previously described^13^.

To investigate the effects of various stressors on ANGPTL2 expression, fully differentiated human adipocytes were incubated in serum-free media for 24 hours, with or without (control) TNF-α (Biosource, Camarillo, CA), IL-1β (R&D Systems, Minneapolis, MN), tunicamycin, thapsigargin, homocysteine, or CoCl_2_ (Sigma-Aldrich, St. Louis, MO), at the concentrations indicated in the Figure legends. Additionally, to examine the effect of a chemical chaperone on stressor-induced ANGPTL2 expression, 4-PBA (Sigma-Aldrich; 500 µM) was added to the culture media 2 hours prior to the stressor treatment. The effect of glucose concentration was also assessed by culturing adipocytes for 24 hours in serum-free media containing either 5.5 mM glucose or 25 mM glucose. ANGPTL2 mRNA in cell lysates and ANGPTL2 protein in culture media were measured using qPCR and ELISA, respectively.

### Culture of 3T3-L1 adipocytes and co-culture with RAW 264.7 macrophages

3T3-L1 cells were cultured and differentiated as previously described^32^. In some experiments, differentiated cells were cultured for 21 days to generate hypertrophic adipocytes containing large lipid droplets. Accumulated triglycerides were visualized by staining with Oil red-O, as previously described^13^. Co-culture was performed using a transwell system. 3T3-L1 cells were grown and differentiated in 6-well dishes, and RAW264.7 macrophages were seeded on polyester membrane inserts (BD Falcon, New York, NC). The cells were co-cultured for 24 hours in serum-free DMEM, with or without tunicamycin (2 µg/mL), and then harvested to determine MMP9, TNF-α, IL-1β (in macrophages), or ANGPTL2 (in adipocytes) mRNA expression.

### Preparation of adipocyte-CM and treatment of macrophages with adipocyte-CM and anti-ANGPTL2 antibody

Next, adipocyte-CM was prepared by collecting the supernatant from the 3T3-L1 adipocytes cultured for 24 hours in the presence of 2 μg/mL tunicamycin. Incubating RAW264.7 macrophages in medium supplemented with adipocyte-CM dose-dependently induced MMP9 mRNA, which reached a maximum with 80% adipocyte-CM (Supplementary Fig. 6a). To confirm the effect of adipocyte-CM, RAW264.7 macrophages were incubated for 16 hours in the medium supplemented with 80% adipocyte-CM or adipocyte media (supernatant from the 3T3-L1 adipocytes cultured without tunicamycin). Then, RAW264.7 macrophages were incubated for 16 hours in medium supplemented with 80% adipocyte-CM in the presence or absence of polyclonal anti-ANGPTL2 antibody (Abcam, Cambridge, UK; 4 μg/mL, determined using a dose-response curve (Supplementary Fig. 6b)) or non-specific IgG (Sigma-Aldrich, 4 μg/mL).

### Statistical analyses

Data are presented as mean ± SD for normally distributed parameters. Non-normally distributed data were log-transformed prior to analysis, to generate a normal distribution. These data are presented as mean ± SE on the original (back-transformed) scale. Student’s paired or unpaired *t*-tests were performed as indicated for pair-wise group comparisons, and ANOVA followed by Tukey’s *post hoc* test was used when >2 groups were compared. Correlation coefficients for two parameters were calculated using Pearson’s correlation. *P* < 0.05 was considered statistically significant. All statistical analyses were performed using SPSS (version 19.0 for IBM, Chicago, IL).

## Electronic supplementary material

Supplementary Information
