# Supplementary material for: Enhanced ANGPTL2 expression in adipose tissues and its association with insulin resistance in obese women
Source: Sci Rep. 2018 Sep 18;8:13976. doi: 10.1038/s41598-018-32419-w (PMC6143523; doi:10.1038/s41598-018-32419-w)
Supplement: Supplementary file 1 — Supplementary Information [file 41598_2018_32419_MOESM1_ESM.pdf]

Supplementary Information

Enhanced ANGPTL2 expression in adipose tissues and its association with insulin resistance in obese women

Jimin Kim[1], Seul Ki Lee[1], Yeon Jin Jang[1*], Hye Soon Park[2*], Jong-Hyeok Kim[3], Joon Pio Hong[4], Yeon Ji Lee[5], Yoon-Suk Heo[6]

[1]Department of Physiology, Cell Dysfunction Research Center, University of Ulsan College of Medicine, Seoul, Korea

[2]Department of Family Medicine, University of Ulsan College of Medicine, Seoul, Korea

[3]Department of Obstetrics and Gynecology, University of Ulsan College of Medicine, Seoul, Korea

[4]Department of Plastic Surgery, University of Ulsan College of Medicine, Seoul, Korea

[5]Department of Family Medicine, Inha University, College of Medicine, Incheon, Korea

[6]Department of General Surgery, Inha University, College of Medicine, Incheon, Korea

	Normal weight	Obesity	Obesity with diabetes
n	32	32	13
Menopause		2	3
Gynecological surgery patients	32	4	
Myoma	21	1	
Leiomyoma	4		
Teratoma			
Cystadenoma			
Others	7	3	
Duration of diabetes			
<1 yr			4
2–9 yr			3
≥10 yr			6
Receiving treatment			
Hypertension			
Beta-adrenergic receptor blocker			3
Angiotensin receptor blocker		2	9
Calcium channel blocker		1	3
Diuretics		2	3
Diabetes			
Metformin			8
Sulfonylurea			3
Insulin			4
Glucosidase inhibitor			2
Dyslipidemia			
Statins			3

Table S1. Clinical characteristics of the non-diabetic obese, obese type 2 diabetic, and normal weight control groups (numbers of patients are shown).

	Before	After	*p* value
BMI (kg/m^2)	38.1 ± 1.4	28.6 ± 1.0	<0.001
Body weight (kg)	106.0 ± 4.9	79.4 ± 3.1	<0.001
Systolic BP (mmHg)	138.7 ± 3.2	118.7 ± 2.8	<0.001
Diastolic BP (mmHg)	83.7 ± 1.8	74.5 ± 2.5	0.009
Glucose (mg/dL)	153.1 ± 15.5	104.0 ± 5.4	0.003
Insulin (μU/mL)	26.9 ± 4.0	8.17 ± 0.79	<0.001
HOMA-IR	10.4 ± 2.0	2.05 ± 0.18	<0.001
Total cholesterol (mg/dL)	186.4 ± 9.5	156.0 ± 7.1	0.006
HDL-cholesterol (mg/dL)	42.7 ± 1.8	45.0 ± 1.8	0.200
LDL-cholesterol (mg/dL)	114.7 ± 7.0	90.0 ± 5.9	0.013
Triglyceride (mg/dL)	231.7 ± 45.5	99.4 ± 12.3	0.005
HbA1c (%)	7.35 ± 0.44	5.71 ± 0.18	<0.001
hs-CRP (mg/dL)	0.61 ± 0.12	0.23 ± 0.08	0.002
Leptin (ng/mL)	28.9 ± 4.4	7.49 ± 1.53	<0.001
Adiponectin (μg/mL)	3.70 ± 0.76	9.07 ± 1.17	<0.001
IL-34 (pg/mL)	0.21 ± 0.02	0.11 ± 0.02	<0.001

Table S2. Obesity index and metabolic parameters measured before and after RYGB surgery in obese subjects. *n* = 23 female patients. Data are shown as the mean ± SE; *P* values were calculated using Student's paired *t*-test. Metabolic parameters were measured before and 5–9 months (average, 7.3 months) after laparoscopic Roux-en-Y gastric bypass (RYGB) surgery.

Gene	Forward primer (5'-3')	Reverse primer (5'-3')
ANGPTL2	GCTGGACCGTCATCCAGAGA	TGCCGTTGTGCCATGTAAAG
ANGPTL2[a]	TGCCATTTGTGTCAACTCCAA	CACGATGCCTCCGTCTACCT
Adiponectin	GGGTGAGAAAGGAGATCCAGGTCTT	AGCGAATGGGCATGTTGGGGA
Leptin	GACATTTCACACACGCAGTCAG	TAGACTGCCAGTGTCTGGTC
TNF-α	GAGCTGAACAATAGGCTGTTCCCA	AGAGGCTCAGCAATGAGTGACAGT
TNF-α[a]	TCTCATGCACCACCATCAAGGACT	TGACCACTCTCCCTTTGCAGAACT
IL-1β	ACAGCTGGAGAGTGTAGATCC	CTTGAGAGGTGCTGATGTACC
IL-1β[a]	AACCTGCTGGTGTGTGACGTTC	CAGCACGAGGCTTTTTTGTTGT
CD68	CTACATGGCGGTGGAGTACAA	ATGATGAGAGGCAGCAAGATGG
CHOP	AGGGAGAACCAGGAAACGGAAACA	TCCTGCTTGAGCCGTTCATTCTCT
TRIB3	GACCGTGAGAGGAAGAAGCTGG	TGCCTTGCCCGAGTATGAGG
MMP9	TGGGCTACGTGACCTATGACAT	GCCCAGCCCACCTCCACTCCTC
MMP9[a]	AAGGCAGCGTTAGCCAGAA	GGAAGACCACAAAAGTCGGC
β-actin	GACGGGGTCACCCACAC	GTGGTGGTGAAGCTGTAGCC
36B4	CCTGAGTGATCTGCAGCTG	CACCTGCTGGATGACCAGC

Table S3. Primer sequences used for real-time qPCR analyses. [a] = mouse primer sequences.

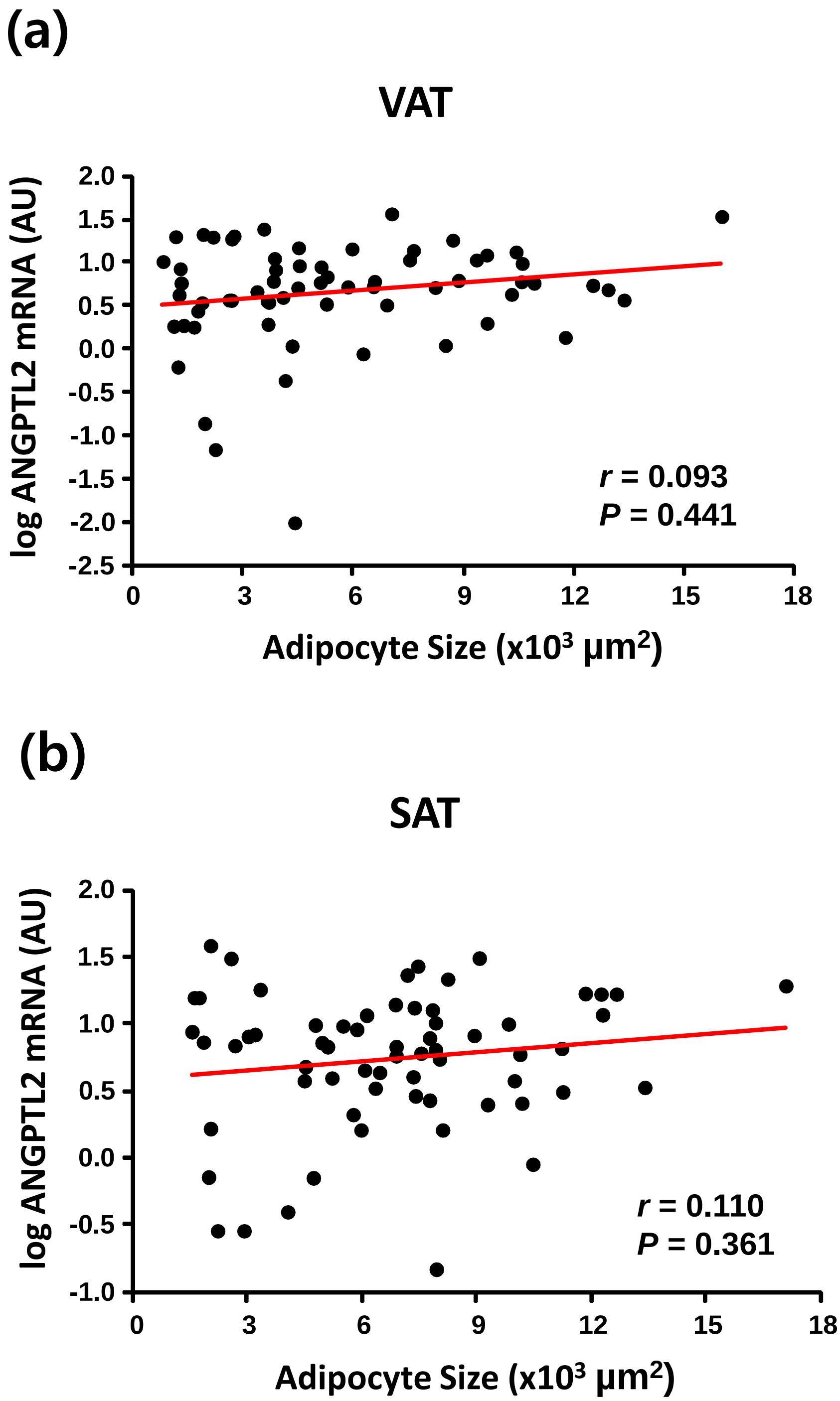

Figure S1. No association between adipose tissue ANGPTL2 mRNA expression and adipocyte size. (a & b) n = 70. The correlation coefficient (r) between changes in measurements was calculated using Spearman's correlation. ANGPTL2 mRNA was log-transformed for statistical analysis and presented in arbitrary units (AU).

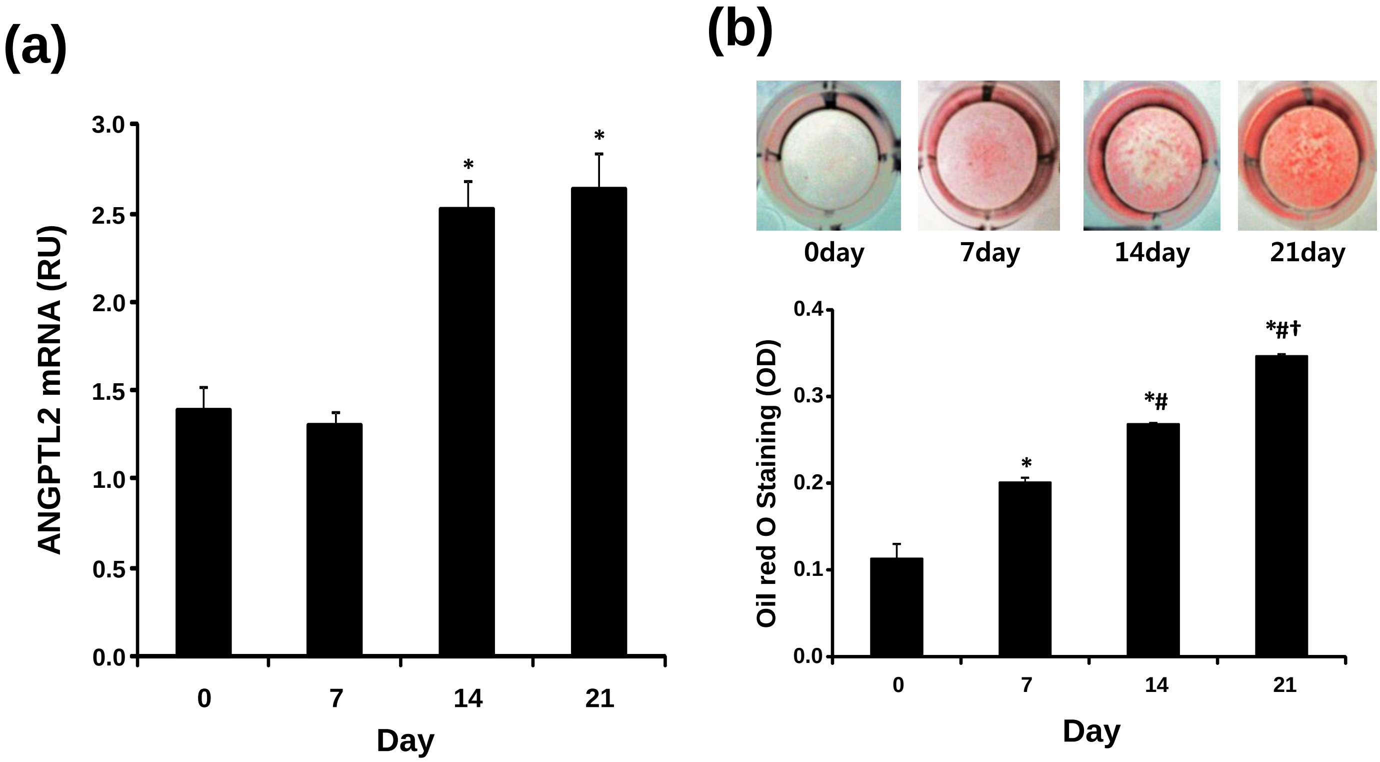

Figure S2. Induction of ANGPTL2 mRNA during adipogenesis. (a) 3T3-L1 preadipocytes were induced to differentiate to adipocytes and maintained up to 21 days. ANGPTL2 mRNA was measured using qPCR ($n = 3$). 36B4 was used as the reference gene. ANGPTL2 mRNA levels are presented in relative units (RU). $^{*}p < 0.01$ *vs.* the day 0 preadipocytes by ANOVA with Tukey test. (b) Accumulation of triglyceride in 3T3-L1 adipocytes. Triglyceride accumulation was visualized by Oil red-O staining and its intensity was determined using a microplate reader (n = 6). $^{*}p < 0.01$ *vs.* the day 0 preadipocytes; $^{\#}p < 0.01$ *vs.* the day 7 adipocytes; $^{\dagger}p < 0.01$ *vs.* the day 14 adipocytes by ANOVA with Tukey test.

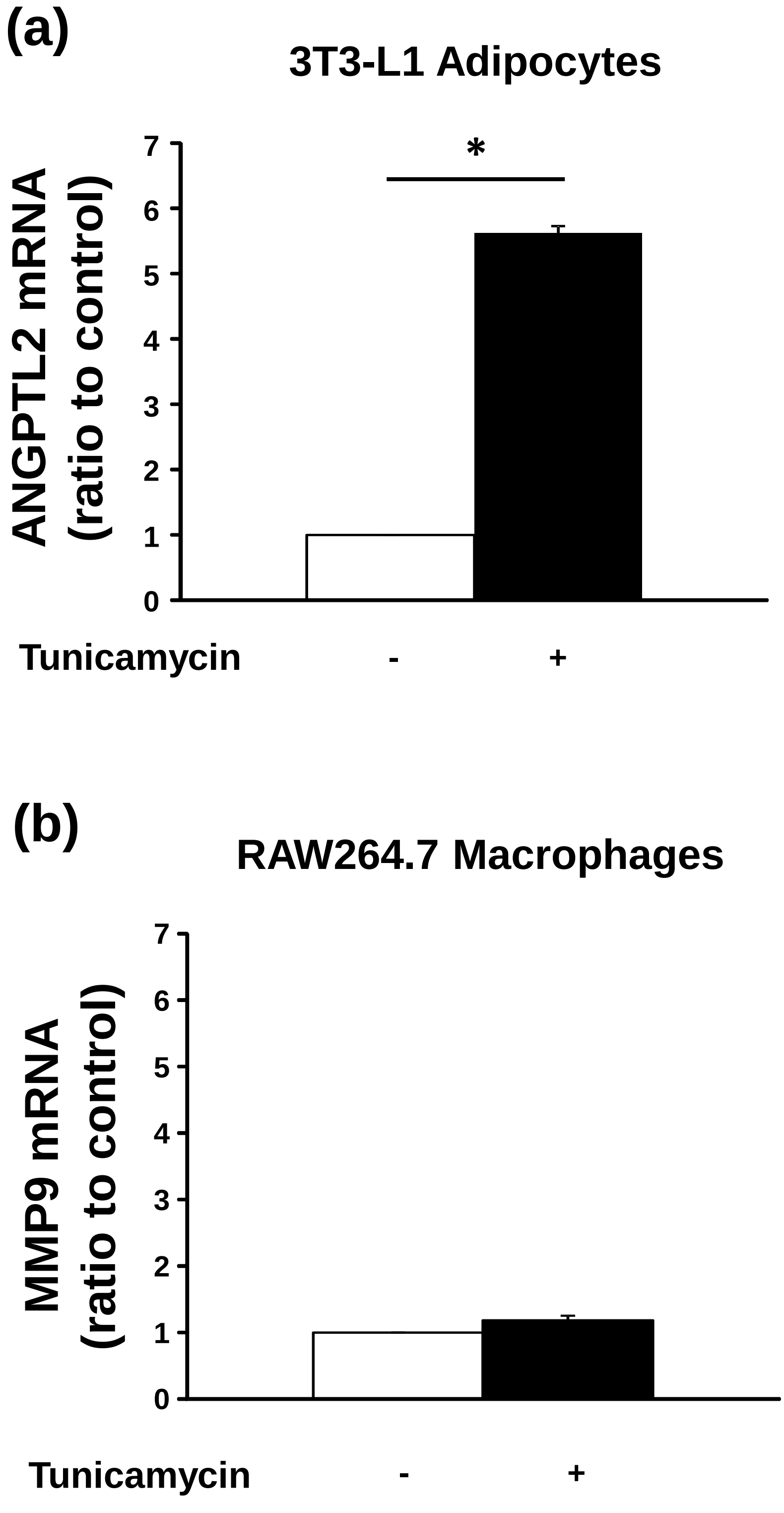

Figure S3. Induction of ANGPTL2 mRNA, but not MMP9 mRNA by tunicamycin. (a & b) Differentiated 3T3-L1 adipocytes and RAW264.7 macrophages were stimulated to tunicamycin (2 μg/mL). ANGPTL2 mRNA in adipocytes (a) and MMP9 mRNA in macrophages (b) were measured using qPCR (n = 3). $^{*}p < 0.01$ *vs*. the cells without treatment of tunicamycin by paired *t*-test.

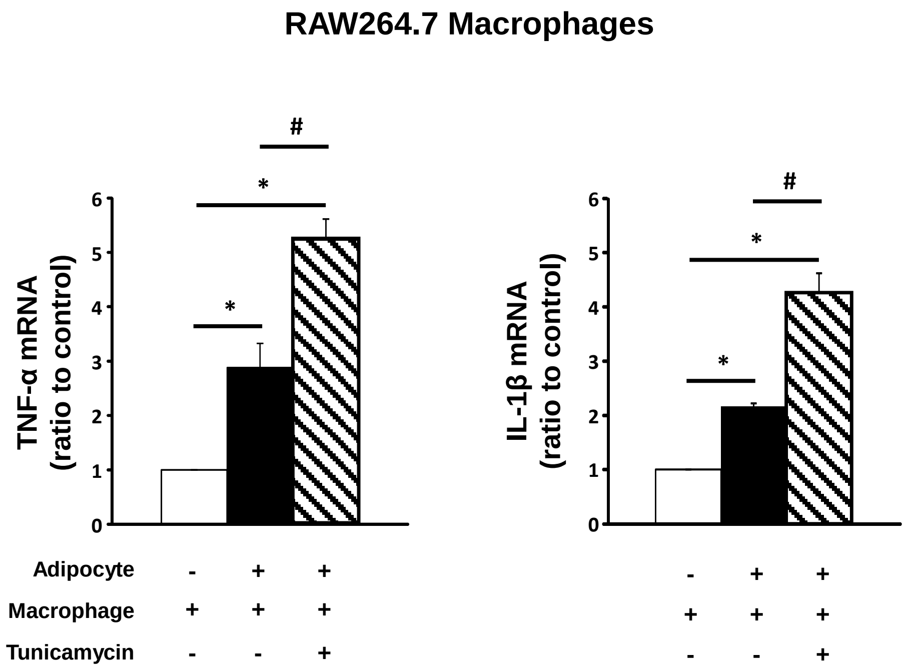

Figure S4. Induction of TNF-α and IL-1β mRNA expression by co-culture of adipocytes and macrophages. RAW264.7 macrophages were co-cultured for 24 hours with differentiated 3T3-L1 adipocytes in the presence or absence of with tunicamycin (2 μg/mL) as described in Fig. 2 legend. Measurement of TNF-α mRNA expression (left panel) and IL-1β mRNA expression (right panel) in macrophages was performed by qPCR (n = 3). $^{*}p < 0.05$ *vs.* macrophages alone, $^{\#}p < 0.05$ *vs.* co-cultured macrophages. The data were analyzed by ANOVA with Tukey test.

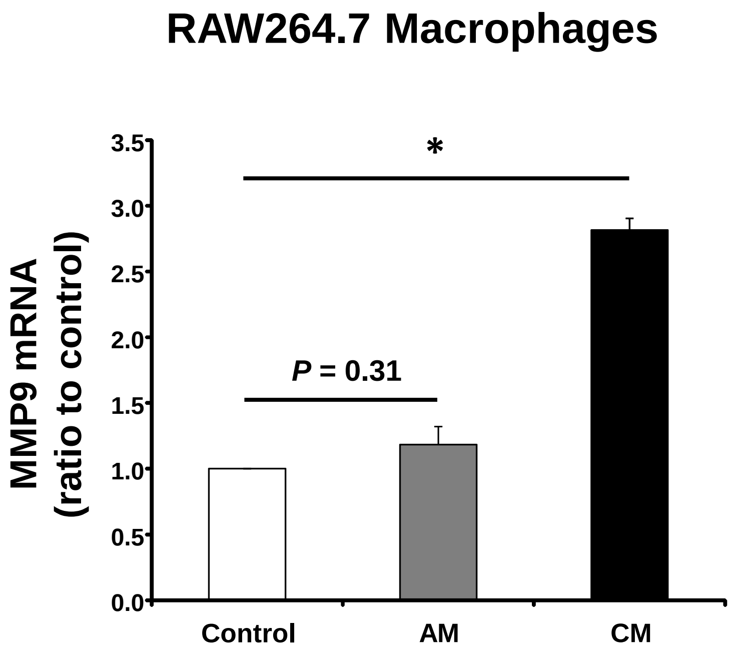

Figure S5. Effects of adipocyte-conditioned media on MMP9 mRNA expression in RAW264.7 macrophages. Adipocyte-conditioned media (CM) was prepared by collecting the supernatant from the 3T3-L1 adipocytes cultured for 24 hours in the presence of 2 μg/mL tunicamycin. RAW264.7 macrophages were incubated for 16 hours in DMEM supplemented with or without (Control) 80% CM or 80% adipocyte media (AM: supernatant from the 3T3-L1 adipocytes cultured without tunicamycin). (n = 3). $^{*}p < 0.05$ *vs.* Control

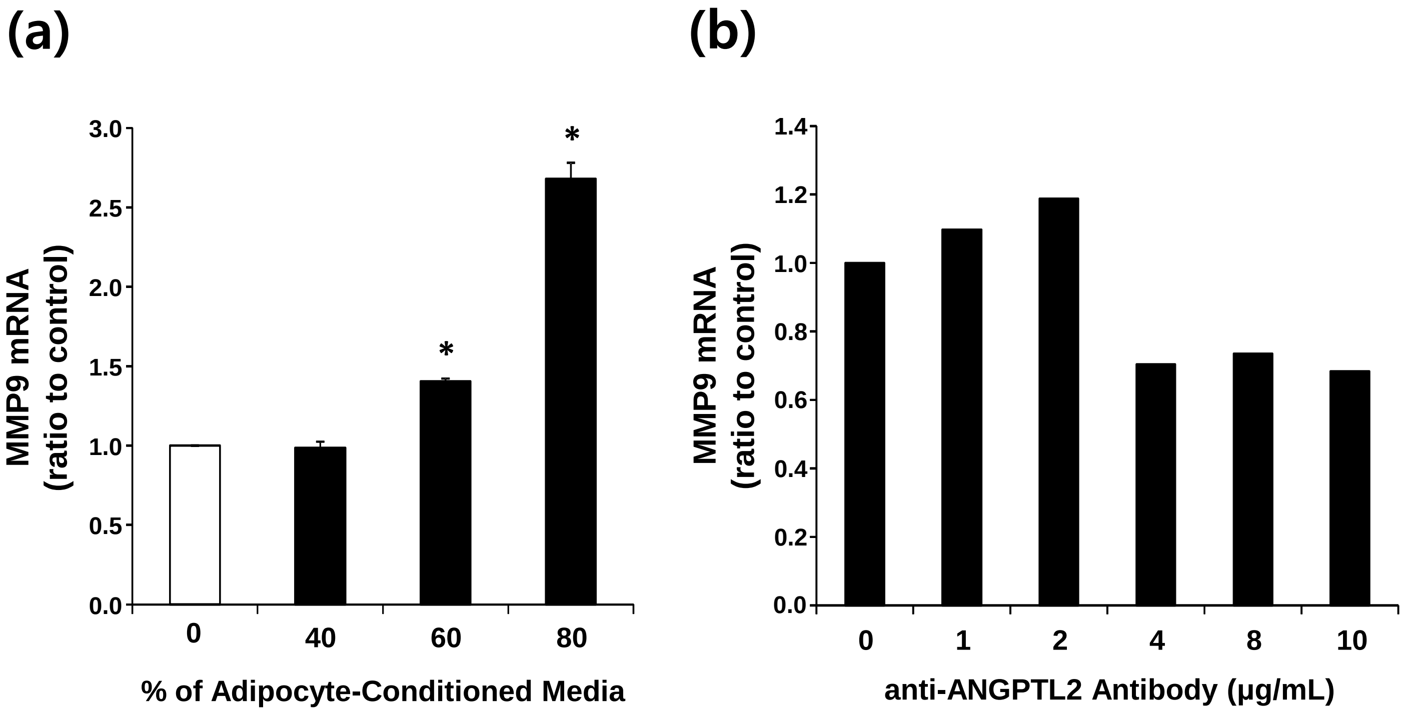

Figure S6. Induction of MMP9 mRNA in RAW264.7 macrophages. (a) Effects of adipocyte-conditioned media (adipocyte-CM). RAW264.7 macrophages were incubated for 16 hours in serum-free DMEM supplemented with from 0% (control) to 80% adipocyte-CM (*n* = 3). Macrophage MMP9 mRNA was measured by qPCR using 36B4 as the reference gene. $^{*}p < 0.05$ *vs*. control by ANOVA with Tukey test. (b) Effects of anti-ANGPTL2 antibody on the induction of MMP9 mRNA by the adipocyte-CM. RAW264.7 macrophages were incubated for 16 hours in serum-free DMEM supplemented with 80% adipocyte-CM, in the presence of varying concentrations (1 – 10 µg/mL) of anti-ANGPTL2 antibody (*n* = 1).
